# Autism, early psychosis, and social anxiety disorder: understanding the role of social cognition and its relationship to disability in young adults with disorders characterized by social impairments

**DOI:** 10.1038/s41398-018-0282-8

**Published:** 2018-10-26

**Authors:** K. L. Pepper, E. A. Demetriou, S. H. Park, Y. C. Song, I. B. Hickie, C. Cacciotti-Saija, R. Langdon, O. Piguet, F. Kumfor, E. E. Thomas, A. J. Guastella

**Affiliations:** 1 Autism Clinic for Translational Research, Brain and Mind Centre, Central Clinical School, Faculty of Medicine, University of Sydney, Camperdown, 2050 Australia; 2 Department of Cognitive Science, Macquarie University, Sydney, Australia; 3 ARC Centre of Excellence in Cognition and its Disorders, Sydney, Australia; 4 The University of Sydney, School of Psychology and Brain and Mind Centre, Sydney, Australia

## Abstract

Impairments in social cognition are believed contribute to disability, particularly for disorders characterized by difficulties in social interaction. There has been little transdiagnostic investigation of this across social cognition domains in young adults. A total of 199 young adults diagnosed with autism spectrum disorder (ASD; *N* = 53), early psychosis (EP; *N* = 51), and social anxiety disorder (SAD; *N* = 64) were compared against neurotypical controls (NT; *N* = 31) on a battery of lower and higher-order and self-report social cognition measures. For both ASD and EP, participants showed impaired performance on all lower-order emotion recognition tasks and one higher-order social cognition test. Self-reports of empathy were reduced in all clinical groups and particularly in ASD. For SAD, despite showing no objective social cognition impairment, self-reported empathy was reduced to the same level as EP. Discriminant analysis revealed that self-reported empathy and lower-order emotion recognition tests provide best capacity to differentiate groups. Regressions predicting disability revealed depression as the strongest predictor across all disability measures. Empathy provided additional predictive value for social disability and social interaction anxiety. Overall, results support a similar social-cognitive development profile across ASD and EP. While self-reported empathy differentiated between groups, discrepancy between objective social cognition test performance and self-reported empathy in the SAD group suggests probable threat-related self-monitoring report biases that likely further influence all group outcomes. As depression and empathy were the most important predictors of disability, regardless of diagnostic group, research is required to explore targeted interventions for difficulties in these domains to reduce disability.

## Introduction

Mental health problems are major contributors to disability burden for young adults in many developed countries^1^. Social impairments are a significant and common feature associated with poor functional outcomes in psychiatric groups^2–4^. Social cognition is believed to contribute to both symptoms and disability, particularly for disorders characterised by social impairment, including autism spectrum disorder (ASD), and psychotic disorders^5^. Social cognition has previously been defined as “the mental operations that underlie social interactions, including perceiving, interpreting, and generating responses to the intentions, dispositions, and behaviours of others”^6^ This can include being able to attend to relevant features of other people’s faces and social scenes^7^, to recognise and label emotions^8^, and to identify and attribute the intention and mental states of others in social scenarios^9,10^. Social cognition skills are often broken into two broad categories reflecting differing brain circuitry processes^11–13^. Lower-order tests involve more rapid and less effortful responses (e.g. emotion recognition), and higher-order tests incorporate reflection, interpretation and deduction when responding to social situations (e.g. theory of mind, attributional accuracy)^14^.

Impairments of social cognition in adult psychotic disorders, including both early psychosis (EP) and fully-developed schizophrenia, are well documented. People with psychotic disorders show deficits on face-processing, emotion-perception, theory of mind and attribution style tasks^15–17^. Such impairments are found to predate the diagnosis of schizophrenia and the onset of other psychotic symptoms^18–20^. The extent of social-cognitive impairment correlates with duration and severity of the schizophrenic illness^20,21^. Similarly, for those with ASD there is a large body of research in children that shows persistent social cognitive impairments^22,23^ and these impairments represent one of the first identifiable markers of the disorder^24^. Research evaluating social-cognitive impairments in adults with ASD is more scant^25,26^, however, although social-cognition impairments have been shown to predict poorer objective social skill^27^.

Current debate of the similarities and differences between ASD and psychotic disorders has highlighted the potential for a broad social development spectrum, where social cognition may provide a broad marker of social disability^25^. Early adulthood typically involves a critical period of life transition for work, personal and family relationships^28^. Transdiagnostic evaluation of social cognition markers in presentations characterised by social impairments in early adulthood is very limited. While some studies show similarities in performance on lower-level emotion recognition between ASD and schizophrenia^29,30^, others suggest that ASD participants may be particularly impaired^31,32^. Previous studies have compared individuals with ASD to fully-developed schizophrenia^30^, which means participants with schizophrenia are often of an older age with a longer history of medical treatments and complications resulting from illness. In terms of the social-cognitive mechanisms that may underpin disability, it has been proposed that those with social-cognitive deficits may find it difficult to integrate and process social-interaction related sensory information effectively, and that this prevents rapid and accurate processing of social information^8,33^. In turn, this breakdown can lead to misinterpretations, mislabelling and potential paranoid thinking. Impairments in social cognition may further reduce the capacity to engage in, and subsequently enjoy, social experiences^15^. The loss of reward associated with social experiences may then exacerbate social withdrawal^15^.

To date, no research has compared performance on social cognition tests between participants with EP and ASD who present to young adult mental health services and report social impairments. Studies have also rarely included comparison groups who are believed to demonstrate significant social difficulties but with no clear social cognitive impairment. Those with social anxiety disorder (SAD) are particularly relevant in this regard as they show significant social withdrawal and disability, despite likely intact social cognition, and are common presentations to young adult mental health settings^34^. Therefore, the first aim of this study was to examine social cognition performance from adults attending a young adult mental health service who have been diagnosed with disorders characterised by social impairment, namely ASD, EP, and SAD, and to compare them with neurotypical controls (NT). We predicted that those diagnosed with a social developmental disorder (ASD and EP) would show significant social cognition impairment in comparison to both SAD and NT participants. The second aim was to determine the discriminant validity of these measures in predicting the social development sub-type. We predicted that both higher-order and lower-order emotion recognition performance would differentiate the social-developmental sub-type. Finally, we aimed to determine whether social cognition performance predicted disability and social interaction concerns above other known factors that influence function (depression and IQ). We hypothesised that social cognition performance would predict greater levels of disability for those diagnosed with a social development disorder (ASD/EP).

## Methods

### Participants

The University of Sydney Ethics Committee approved the research protocol for this study (Project number: 2013/352). Informed written consent was obtained directly from each participant prior to their inclusion in the study. A cohort of young adults (*N* = 199, Age: *M* = 23 years 1 month, range 16–46 years, with 89% in range 16–30 years) sequentially presented for treatment and/or social skills development at the Autism Clinic for Translational Research (ACTr) and *Headspace* Brain and Mind Centre clinics were recruited into the study. Clinical participants met primary diagnostic criteria for ASD (*N* = 53), EP (*N* = 51), or SAD (*N* = 64). Research qualified clinicians at the ACTr assessed participants and made formal diagnoses based on standardised diagnostic instruments and clinical case files. For those diagnosed with ASD, participants met clinical cut-off on the Autism Diagnostic Observation Schedule—2nd edition (ADOS-2)^35^ and a clinical interview assessing DSM-V criteria. Participants meeting criteria for SAD completed the Anxiety Diagnostic Interview Schedule (ADIS-IV/V)^36,37^. Participants were screened for psychotic symptoms, and any reporting psychotic symptoms were excluded from the ASD and SAD groups. Participants at clinical interview or at referral that were suspected of showing any ASD like symptoms were also screened on the ADOS. Participants meeting criteria for EP completed the Structured Clinical Interview for DSM-IV Axis I Disorders (SCID-I)^38^. The NT participants (*N* = 31) were recruited separately through advertising on university websites. Full scale IQ was estimated using either the two subtest version of the Wechsler Abbreviated Scale of Intelligence (WASI)^39^ for the EP group (who were assessed under a slightly different protocol than the other groups), or the Wechsler Test of Adult Reading (WTAR)^40^ for the remaining groups. All participants were screened and excluded from the study if they had an intellectual disability (IQ < 70), a neurological condition, or current substance dependence. The NT participants were excluded if they reported a mental health diagnosis (past or current) or of they scored above cut-offs on the Depression, Anxiety and Stress Scale-(DASS-21)^41^, the Social Interaction Anxiety Scale (SIAS)^42^, or the short-form of the Autism Quotient (AQ-10)^43^.

### Measures

The study utilised a battery of tests to measure objective performance in either “lower order” (emotion recognition) or “higher order” (theory of mind) domains of social cognition. A self-report measure of social cognition (Empathy Quotient) was also included so that the participants’ self-perception of their social cognition abilities can be compared with their actual performance. Self-report measures of symptom severity, mood and disability were also completed. Detailed descriptions of the measures are presented in Supplementary Table 1.

### Emotion recognition

The Reading the Mind in the Eyes (RMET) test assesses the participant’s ability to recognise a range of emotions from photographs of the eye region of human faces^44^. The Facial Expressions of Emotions: Stimuli and Tests (FEEST) is a test of facial emotion recognition in which emotions must be identified for photographs of whole faces^45^. The Movie Stills task involves identifying emotion in photographs of complex scenes, firstly with the faces of the scene participants blanked out, and secondly with the faces visible. It assesses emotion recognition from purely contextual cues (“no face” condition) and by using both facial expressions and contextual cues to determine emotion (“face” condition)^46^. In all these measures, higher scores indicate higher levels of emotion recognition.

### Theory of mind

The Faux Pas Recognition Task assesses the participants’ understanding of socially awkward situations and their appreciation of the emotional impact of a statement on a listener^47^. The False Belief Picture Sequencing Task (FBPST) assesses the participants’ ability to identify whether a person has acted on a false belief and is a classic theory of mind measure^48^. The Cambridge Behaviour Scale Abbreviated Empathy Quotient (EQ) is a self-report measure that assesses affective empathy (e.g. “Seeing other people cry does not really upset me”) or cognitive empathy (e.g. “I can easily work out what another person might want to talk about”) in social situations or relationships^49,50^.

### Symptom severity, mood and disability measures

The Social Interaction and Anxiety Scale (SIAS) requires participants to rate how much anxiety they typically experience in a range of social interactions^42^. The Depression Anxiety Stress Scale (DASS-21), reflects levels of depression, anxiety and stress as rated by the participant for the previous week^41^. The World Health Organisation Disability Assessment Schedule 2 (WHODAS-2.0), assesses difficulty in a variety of everyday circumstances grouped into six domains, including understanding and communicating, getting around, self-care, getting along with people, life activities (home/school/work), and participation in society^51^.

### Data analysis

Data relating to the participants’ demographic characteristics, performance and self-report measures of social cognition and general well-being questionnaires were statistically analysed using the IBM SPSS Statistics Version 24 analysis program. Total scores for each of the measures were calculated from each participant’s database records according to the standard scoring algorithms established for each measure. Missing data for all variables except the WHODAS measures were imputed using the mean total score. WHODAS scores were included in the regression only for those participants who had full data, in accordance with the WHODAS scoring manual instructions (See Supplementary Table 2 for missing data reports).

Univariate (ANOVA) and multivariate analysis of variance (MANOVA) were completed to examine for differences between the clinical and NT groups on social cognition measures and disability, followed by Gabriel or Games-Howell post hoc comparison tests with Bonferroni corrections to take into account unequal sample sizes and unequal variances with capacity to detect moderate effect sizes. Discriminant analysis was used to determine which social cognition measures best discriminated between diagnostic groups. Multiple regression (MR) analyses examined the predictive value of the diagnosis, IQ, depression and social cognition measures on three different measures of disability, as assessed by the WHODAS overall score, WHODAS Domain 4 (Getting Along with People) and SIAS.

## Results

### Demographics

Demographic characteristics are presented in Table 1. No group differences were present for sex (*χ*^2^_(3, *N* = 199)_ = 4.12, *p* > 0.05) and age (*F*_(3,93.6)_ = 2.46, *p* > 0.05). A one-way ANOVA showed a significant difference between groups in IQ, (*F*_(3, 94.8)_ = 8.203, *p* < 0.001) and DASS Depression (*F*_(3, 16.2)_ = 58.1, *p* < 0.001). Although all groups remained in the normal IQ range, both the SAD and NT groups showed slightly higher IQ than EP. Depression was also greater in SAD and ASD, followed by EP, and all were more depressed than NT.

**Table 1:** Demographic characteristics of participants with a primary diagnosis of autism spectrum disorder (ASD), early psychosis (EP), social anxiety disorder (SAD), and neurotypical control (NT) participants

Primary diagnosis	ASD_a_ Group		EP_b_ Group		SAD_c_ Group		NT_d_ Group		Statistical analysis						
Sex	N	%	N	%	N	%	N	%	Pearson Chi-Square						
Male	35	66.0	36	70.6	34	53.1	19	61.3	χ^2^_(3)_ = 4.12, p > 0.05						
**Demographic characteristics**	**Mean**	**SD**	**Mean**	**SD**	**Mean**	**SD**	**Mean**	**SD**	**ANOVA**	**Post-hoc Multiple Comparisons (*p*** = 0.05)					
N	53		51		64		31			a *vs* b	a *vs* c	a *vs* d	b *vs* c	b *vs* d	c *vs* d
Age (years)	23.92	*7.40*	21.75	*4.38*	22.67	*5.99*	24.77	*6.08*	F_(3,93.6)_ = 2.46	ns	ns	ns	ns	ns	ns
IQ	106.51	*9.04*	102.24	*14.10*	111.39	*7.01*	110.84	*7.02*	F_(3,94.8)_ = 8.20***	ns	*	ns	***	**	ns
Depression	22.90	*12.47*	16.43	*12.72*	24.38	*11.22*	4.71	*5.36*	F_(3, 106.2)_ = 58.12***	*	ns	***	**	***	***

*p < .05; **p < .01; ***p < .001

### Medication and drug use

Data on psychotropic medication and recreational drug use are summarised in Supplementary Tables 3 and 4.

### Social cognition performance measures

#### Emotion recognition

**Fig. 1:**
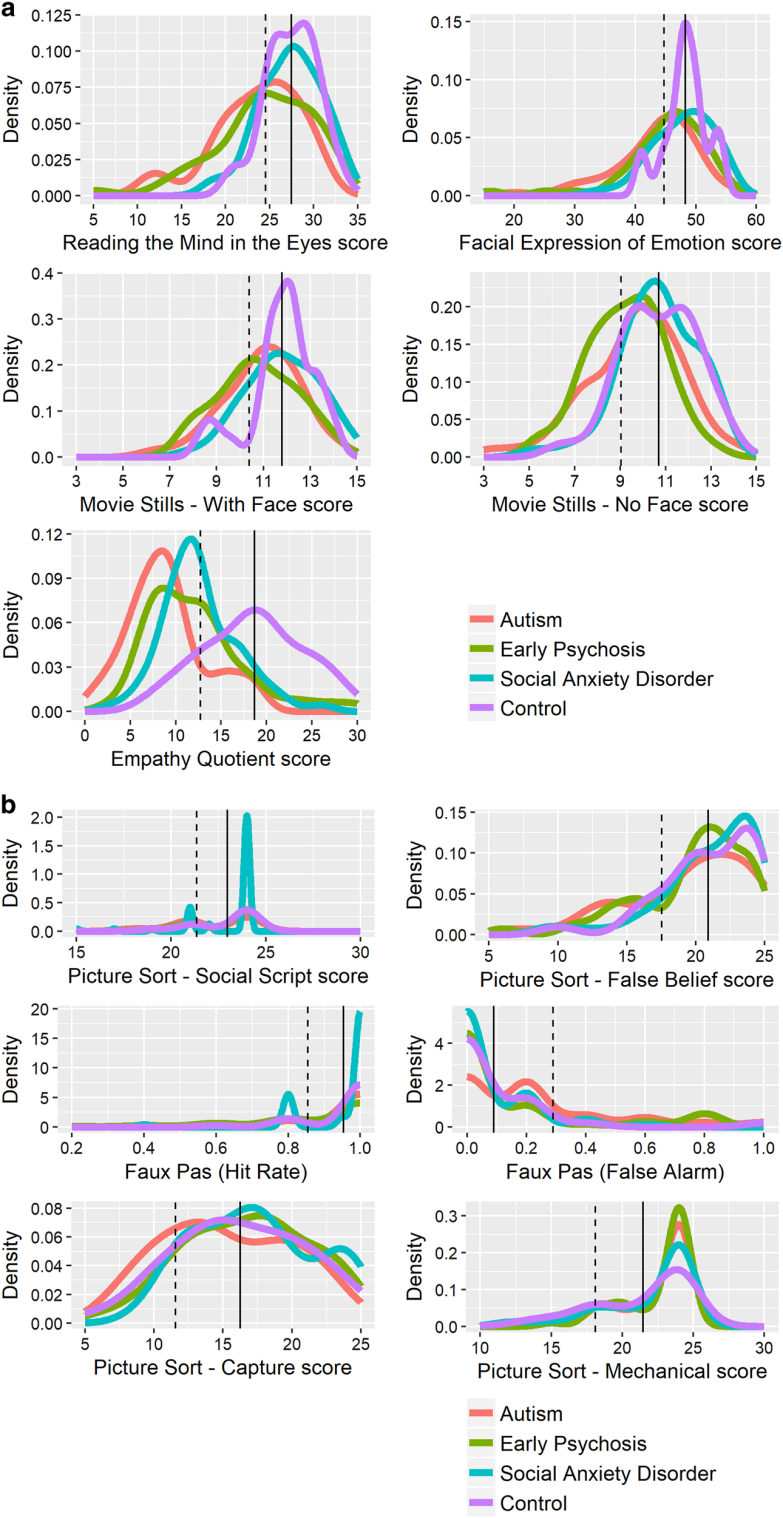
Density plots of social cognition measures for autism spectrum disorder, early psychosis, social anxiety disorder, and neurotypical controls. The solid and dashed vertical lines represent the mean of the neurotypical control group and performance that is one standard deviation below the mean of the neurotypical control group, respectively

Descriptive statistics and the results of MANOVA for the social cognition lower-order measures are summarised in Table 2 and density plots in Fig. 1. The overall MANOVA for diagnosis was significant (Pillai Trace: *F*_(12,582)_ = 3.60, *p* < 0.001) for the lower-order social cognition measures (RMET, FEEST, Movie Stills_No-Face_ and Movie Stills_Face_). Univariate analyses of variance revealed significant differences between groups for the emotion recognition measures. Overall, follow-up tests showed similar performance between SAD and NT and, separately, between EP and ASD. Scores were generally significantly different between these SAD/NT and EP/ASD groups on follow-up tests, although the total FEEST score did not reach significance between EP and SAD groups specifically (*p* = 0.08).

**Table 2:** Descriptive statistics (mean, SD, *n*), and significance tests (F) comparing social cognition measures between participants with autism spectrum disorder (ASD), early psychosis (EP), social anxiety disorder (SAD), and typically developing controls (NT)

Primary diagnosis	ASD_a_ group (*N* = 53)		EP_b_ group (*N* = 51)		SAD_c_ group (*N* = 64)		NT_d_ group (*N* = 31)		ANOVA	Post hoc pairwise comparisons					
	Mean	SD	Mean	SD	Mean	SD	Mean	SD		a vs b	a vs c	a vs d	b vs c	b vs d	c vs d
**Social cognition self-report measure**															
EQ total	8.99	*4.62*	12.16	*5.47*	12.64	*4.15*	18.49	*5.45*	*F*_(3,195)_ = 25.87***	**	***	***	ns	***	***
**Social cognition (lower order) performance measures**									*F*_(12,582)_ = 3.60***						
RMET	23.58	*4.93*	24.39	*5.63*	27.33	*3.56*	27.45	*2.91*	*F*_(3,195)_ = 9.81***	ns	***	**	**	**	ns
FEEST	43.44	*6.77*	44.98	*7.22*	47.88	*5.07*	48.27	*3.54*	*F*_(3,195)_ = 7.30***	ns	**	**	ns	*	ns
Movie Stills_No Face_	9.62	*2.11*	9.25	*1.68*	10.66	*1.70*	10.70	*1.66*	*F*_(3,195)_ = 8.06***	ns	**	*	***	**	ns
Movie Stills_Face_	10.94	*1.66*	10.76	*1.69*	11.81	*1.58*	11.80	*1.40*	*F*_(3,195)_ = 5.98**	ns	*	ns	**	*	ns

**Social cognition (higher-order) performance measures**									*F*_(12,582)_ = 2.38**	**a vs b**	**a vs c**	**a vs d**	**b vs c**	**b vs d**	**c vs d**
Picture Sequencing_Mechanical_	22.17	*2.81*	22.22	*3.39*	21.94	*3.25*	21.45	*3.36*	*F*_(3,195)_ = 0.44	ns	ns	ns	ns	ns	ns
Picture sequencing_Capture_	15.30	*4.73*	16.76	*4.55*	17.33	*4.39*	16.26	*4.69*	*F*_(3,195)_ = 1.99	ns	ns	ns	ns	ns	ns
Picture sequencing_Social Script_	21.87	*2.65*	22.63	*2.37*	23.11	*1.81*	22.97	*1.62*	*F*_(3,195)_ = 3.42	ns	*	ns	ns	ns	ns
Picture sequencing_False Belief_	19.42	*4.49*	19.78	*3.98*	20.92	*3.49*	20.90	*3.38*	*F*_(3,195)_ = 1.99	ns	ns	ns	ns	ns	ns
Faux Pas_Hits_	0.935	*0.134*	.878	*0.192*	0.947	*0.106*	0.954	*0.100*	*F*_(3,195)_ = 2.96*	ns	ns	ns	ns	ns	ns
Faux Pas_False Alarms_	.223	*0.265*	0.141	*0.260*	0.073	*0.128*	0.090	*0.199*	*F*_(3,195)_ = 5.04**	ns	**	ns	ns	ns	ns

*p < 0.05; **p < 0.01; ***p < 0.001

#### Higher-order social cognition

Descriptive statistics and MANOVA results for the social cognition higher-order measures are summarised in Table 2 and density plots in Fig. 1. A significant difference was found between groups on higher-order social cognition measures (Pillai’s trace *F*_(12,582)_ = 2.38, *p* = 0.005). Examination of post hoc outcomes revealed only the false alarm rate on the Faux Pas test was significantly different between groups, *F*_(3,195) _= 5.04, *p* = 0.002, while both the social script subscale of the FBPST, *F*_(3,195)_ = 3.42, *p* = 0.03, and the Faux Pas hit rate, *F*_(3, 195)_ = 2.96, *p* = 0.03, were at trend levels following Bonferroni correction. SAD participants showed a lower Faux Pas false alarm rate than ASD. One-way ANOVA tests on the non-social subscales of the False Belief Picture Sequencing Test confirmed that there was no difference between groups on those subscales of the FBPST, including the mechanical or capture subscales (See Table 2 and Fig. 1).

Analyses was repeated with both depression and IQ as covariates. The MANOVA results remained significant and similar in magnitude when these covariates were included. Analyses were also repeated with medication status overall and also with anti-depressants and anti-psychotics separately included as additional independent variables, and there were no significant interaction effects between diagnosis and medication status.

### Social cognition self-report measures

#### Empathy

EQ scores between the diagnostic groups were compared using a one-way ANOVA (see Table 2 and Fig. 1) which showed a significant difference. The NT group reported higher empathy compared to the clinical groups. In addition, the ASD group reported lower empathy than all other groups.

### Discriminant validity

**Fig. 2:**
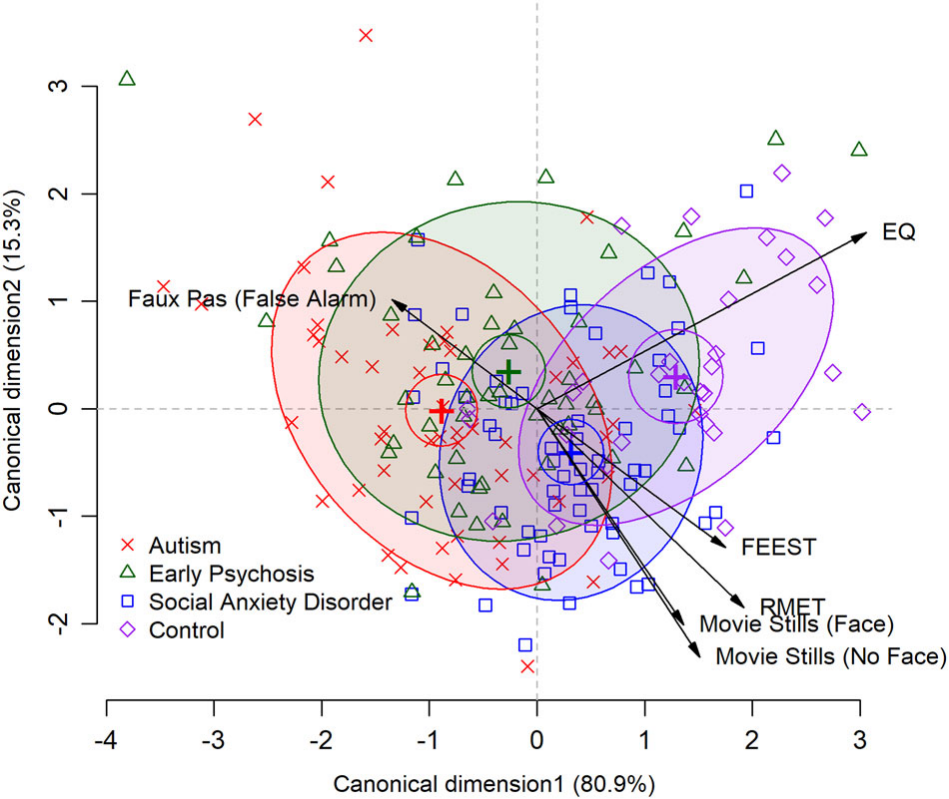
Discriminant function plot of significant social cognition variables that differentiate between the clinical and neurotypical control groups .

A discriminant analysis of the significant social cognition variables was run to differentiate group assignment on the significant variables identified above (EQ, RMET, FEEST, Movie Stills Face and No Face, and the Faux Pas False Alarm Rate). The analysis provided two significant discriminant functions (Fig. 2). The first one accounted for 80.9% of the variance, canonical *R*^2^ = 0.34 and the second accounted for 15.4% of the variance, canonical *R*^2^ = 0.09. The third factor was not significant and accounted for only 0.38% of the variance, canonical *R*^2^ = 0.02. In combination, these first two discriminant functions significantly differentiated the diagnostic groups, ˄ = 0.58, *χ*^2^ (18) = 105.00, *p* < 0.001; ˄ = 0.89, *χ*^2^ (10) = 23.14, *p* = 0.01. The correlations between the outcomes and the discriminant functions indicated that the EQ (*r* = 0.84) and the FEEST (*r* = 0.43) loaded onto the first function. The second function showed high correlations with the RMET (*r* = 0.54), Movie Still _Face_ (*r* = 0.57) and Movie Still _No Face_ = (*r* = 0.66) (See Fig. 2).

### Effects of social cognition predictors on health and lifestyle disability measures (WHODAS—overall rating)

A MR examined the relationship of the predictors of diagnosis, IQ, depression and social cognition performance measures on the WHODAS overall rating (Table 3a). The overall model was significant across the study cohort, *F*_(11,172)_ = 14.39, *p* < 0.001) and explained 44.6% of the total variance. The only significant predictor was depression and diagnostic groups. Follow-up analysis showed that there was no interaction between diagnosis and depression, suggesting that the model contributed the same across diagnosis.

**Table 3:** Multiple regression analyses showing the relationship of social cognition, IQ, depression, and clinical group to self-rated disability and social anxiety

	*B*	SE *B*	*β*
*3a: WhoDAS overall rating*			
Constant	34.554	15.542	
IQ	−0.065	0.135	−0.033
DASS depression	0.659	0.099	0.455***
RMET	0.438	0.318	0.109
FEEST	−0.357	0.212	−0.120
Movie Still_No Face_	−0.213	0.660	−0.021
Movie Still_Face_	−0.756	0.763	−0.065
Faux Pas_False Alarm_	5.704	5.279	0.068
EQ	−0.367	0.225	−0.110
ASD group	11.310	4.256	0.269**
EP group	15.340	4.004	0.323***
SAD group	11.271	3.796	0.281**
Model *R*^2^ = 0.446			
*3b: WhoDAS Domain 4 “Getting along with people”*			
Constant	31.374	23.532	
IQ	0.103	0.201	0.035
DASS depression	0.948	0.148	0.441***
RMET	0.066	0.476	0.011
FEEST	0.129	0.318	0.029
Movie Still_No Face_	−0.642	0.987	−0.043
Movie Still_Face_	−0.544	1.141	−0.031
Faux Pas_False Alarm_	5.190	7.892	0.041
EQ	−1.497	0.336	−0.303***
ASD group	2.933	6.362	0.047
EP group	6.758	5.985	0.096
SAD group	11.673	5.675	0.196
Model *R*^2^ = 0.438			
*3c: Social Interaction Anxiety Scale*			
Constant	27.74	12.59	
IQ	0.062	0.106	0.033
DASS depression	0.660	0.082	0.443***
RMET	−0.145	0.258	−0.035
FEEST	−0.223	0.181	−0.071
Movie Still_No Face_	0.247	0.558	0.024
Movie Still_Face_	0.947	0.646	0.080
Faux Pas_False Alarm_	−9.351	4.410	−0.107
EQ	−0.833	0.190	−0.238***
ASD group	6.383	3.663	0.144
EP group	−0.914	3.330	−0.020
SAD group	16.117	3.253	0.385***
Model *R*^2^ = 0.605			

*p < 0.05, **p < 0.01, ***p < 0.001

### Effects of social cognition predictors on getting along with people (WHODAS – Domain 4)

A second MR examined the relationship of the predictors of diagnosis, IQ, depression and social cognition performance measures on the WHODAS ratings for Domain 4: Getting Along with People (Table 3b). The overall model was significant across the study cohort, *F*_(11, 172_ = 13.95, *p* < 0.001) and explained 43.8% of the total variance, with significant predictors being depression and EQ. Neither depression or EQ interacted with diagnosis to predict social disability.

### Effects of social cognition predictors on the SIAS

The final MR examined the relationship of the predictors of diagnosis, IQ, depression, social cognition performance measures and social cognition self-report measures on social interaction anxiety (SIAS) (Table 3c). The model was significant across the study cohort (*F*_(11, 187)_ = 28.53, *p* < 0.001), and explained 60.5% of the total variance. Significant predictors were SAD diagnosis, depression and EQ, while there was a trend for the Faux Pas false alarm rate. For these significant predictors, interactions with diagnosis were entered in follow-up analyses with each interaction effect run separately. Results did not show interactions with diagnosis for significant predictors.

## Discussion

As far as we are aware, this is the first study to compare higher-order and lower-order social cognition performance in young adults diagnosed by disorders characterized by social impairment who present to youth mental health services, including EP, ASD, and SAD. Results revealed that individuals diagnosed with ASD and EP showed greater impairments in lower and higher-order social cognition in comparison to both NT and those diagnosed with SAD. These participants specifically showed reduced performance on all lower-order emotion recognition tests and on the false alarm rate of the Faux Pas test. Interestingly, this study showed that all clinical groups reported impairments on the EQ, even though SAD participants showed no evidence of objective deficits in social cognition. Discriminant analysis was then conducted to determine which of the social cognition variables discriminated between diagnostic groups. Results indicated that the first function, where the EQ and FEEST loaded most heavily, provided best capacity to differentiate between groups. The second function, consisting of all other lower-order emotion recognition tests (RMET and Movie Stills), provided additional differentiating value. The third aim of this study was to determine the degree that performance on these social cognition measures predicted disability, social disability and social interaction anxiety. Our results showed that across the population, the most important predictor of all types of disability was depression. The only social cognition measure to predict social interaction anxiety and social disability was EQ, and this was shown across all clinical groups. Surprisingly, there was no interaction with diagnosis to support the view that social cognition might predict disability differently in those social development disorders.

The results of this study highlight the particular utility of lower-order emotion recognition tests in differentiating between social development disorders and NTs^30^. Not only was the group ASD and EP performance significantly lower on all tests, but in combination with the EQ, they provided best capacity to differentiate disorders. Emotion recognition has a long history of importance as a fundamental building block to social cognition and social skill. Its role in core social and emotional brain networks is well established^11,12^. Further studies are now required across broader cohorts to determine the potential of emotion recognition tests as a screener for social development concerns. While the higher-order tests we chose are well-established, impairment has previously been principally shown in psychotic populations. Some evidence in psychotic populations suggests that higher-order performance might deteriorate with age^52^. Application in this young population might contribute to the absence of impairment on these tests. Longitudinal studies tracking social cognitive development are now required to determine whether this deterioration might be specific to psychotic populations or a feature of other neurodevelopmental disorders such as ASD.

It is likely that differences in brain circuitry integrity underlie the differences in social cognition performances found between the neurodevelopmental groups compared in the present study. Lower level social cognition is typically associated with activity/integrity of brain regions that include the amygdala, insula, ventromedial prefrontal cortex and the anterior temporal poles^11,12^. In contrast, higher level social cognition performance typically relies on the integrity of posterior regions, specifically the temporoparietal junction^13^. Here, our findings of a relatively preserved higher level social cognition in the context of reduced lower-level social cognition capacity would suggest a disturbance of anterior brain regions in ASD and EP. Indeed, abnormalities in frontotemporal and amygdala brain regions have been associated with socioemotional dysfunction in ASD^53,54^. Further, a recent pilot study reported decreased functional connectivity between the ventromedial prefrontal cortex and fusiform cortex in EP compared to NTs^55^. Undoubtedly, additional research will be needed to identify the neural underpinnings of social cognition performance, as these may eventually serve as biomarkers of social development disorders.

Regarding the EQ, the measure was found to not only best differentiate clinical disorders from NT but also to predict social disability and social interaction anxiety. Interestingly, individuals with SAD showed intact performance on all objective social cognition measures but reported impaired empathy. Such results may reflect a combination of self-monitoring biases and reflection of social difficulty of real life scenarios, which in SAD is underpinned by hypervigilance and avoidance of perceived threat^5^. While the study provides further support for negative self-evaluation in the context of SAD^56^, they highlight the non-specificity of the EQ in tapping into social problems identified by clinical participants. It is therefore likely that self-monitoring biases could also influence reports of those with ASD and EP and future studies should include independent reports and objective social performance measures to understand this relationship.

All of our young adult clinical groups also reported significant disability impairment compared to NTs as measured by the WHODAS. In our cohort, depression was the most important predictor of disability, social disability and social interaction anxiety. Depression’s role in increasing disability in youth cohorts is increasingly recognised^5,57^. Depression likely disables engagement with social support networks, through loss of motivation and reward, to impact on functional outcomes. These results suggest opportunities to target depression and empathy with the hope of improving disability across clinical groups.

Our study has several limitations. Firstly, this battery does not include other well-established constructs and measures of social cognition, including interoceptive awareness, affective and cognitive empathy, and recently developed multidimensional tests in naturalistic settings^14^. Related to this, our higher-order tests are well accepted, and they have been applied to EP populations previously^52^, but they show less variability in this population. Other higher-order tests deserve further investigation given the findings of this study. We note that the WHODAS was our primary measure of disability. While this measure is currently regarded internationally as the most important measure of disability, it is based on self-report. It is possible that report biases contribute to the relationship between our self-report measures and our measure of disability, and objective measures are required. We also note this is a cross-sectional study and future research using longitudinal designs will be needed to understand how change across time influences disability. It also needs to be noted that participants in the ASD and EP groups were not excluded if they had comorbid SAD, nor were they routinely diagnostically assessed for SAD. This study was primarily concerned with the neurodevelopmental profiles of those with ASD and EP, in comparison to those with SAD who did not show any evidence of these neurodevelopmental disorders but also clear social difficulties. Future studies may wish to investigate possible differences in social cognition between individuals in these neurodevelopmental groups with and without comorbid social anxiety. In addition, many of the participants in the diagnostic groups were being treated with psychotropic medications, and some recreational alcohol and drug use was also present in all groups. While our results did not show significant interactions with medication status, we were unable to control entirely for medication and also illicit drug use in this study. Future studies should investigate these effects across these clinical cohorts. Finally, participants with intellectual disability were excluded from this study due to measurement completion requirements. Studies need social cognitive tasks that do not rely on language and intellectual capacity to engage a broader affected population.

In conclusion, this study shows that individuals diagnosed with disorders characterised by social development impairments, ASD and EP, demonstrate impairments on lower-order social cognition and these tests, along with the EQ, provide best capacity to differentiate between diagnostic groups. Further research is now required to understand the neurocircuitry of this impairment in adulthood. In regards to disability, however, depression and self-reported EQ provide the most important predictors across these psychiatric populations. These results suggest opportunities to target depression and empathy with the hope of improving social disability across these clinical groups.

## Electronic supplementary material

Supplementary Information

## References

[1] Trautmann S, Rehm J, Wittchen HU (2016). The economic costs of mental disorders. EMBO Rep..

[2] Stein MurrayB, Yin, Kean M (2000). Disability and Quality of Life in Social Phobia: Epidemiologic Findings. Am. J. Psychiatry.

[3] Wiersma D (2000). Social disability in schizophrenia: its development and prediction over 15 years in incidence cohorts in six European centres. Psychol. Med..

[4] Howlin P, Magiati I (2017). Autism spectrum disorder: outcomes in adulthood. Curr. Opin. Psychiatry.

[5] American Psychiatric Association. (2013). Diagnostic and Statistical Manual of Mental Disorders.

[6] Green MF (2008). Social cognition in schizophrenia: an NIMH workshop on definitions, assessment, and research opportunities. Schizophr. Bull..

[7] Williams LM (2003). Emotion perception in schizophrenia: an eye movement study comparing the effectiveness of risperidone vs. haloperidol. Psychiatry Res..

[8] Kee KS, Horan WP, Wynn JK, Mintz J, Green MF (2006). An analysis of categorical perception of facial emotion in schizophrenia. Schizophr. Res..

[9] Premkumar P (2008). Misattribution bias of threat-related facial expressions is related to a longer duration of illness and poor executive function in schizophrenia and schizoaffective disorder. Eur. Psychiatry.

[10] Kettle JW (2008). Impaired theory of mind in first-episode schizophrenia: comparison with community, university and depressed controls. Schizophr. Res..

[11] Kennedy DP, Adolphs R (2012). The social brain in psychiatric and neurological disorders. Trends Cogn. Sci..

[12] Kumfor F, Irish M, Hodges JR, Piguet O (2013). Discrete neural correlates for the recognition of negative emotions: Insights from frontotemporal dementia. PLoS ONE.

[13] Samson D, Apperly IA, Chiavarino C, Humphreys GW (2004). Left temporoparietal junction is necessary for representing someone else’s belief. Nat. Neurosci..

[14] Turner R, Felisberti FM (2017). Measuring mindreading: a review of behavioral approaches to testing cognitive and affective mental state attribution in neurologically typical adults. Front. Psychol..

[15] Penn DL, Sanna LJ, Roberts DL (2008). Social cognition in schizophrenia: an overview. Schizophr. Bull..

[16] Langdon R (1997). Defective self and/or other mentalising in schizophrenia: a cognitive neuropsychological approach. Cogn. NeuroPsychiatry.

[17] Langdon R, Coltheart M, Ward PB, Catts SV (2002). Disturbed communication in schizophrenia: the role of poor pragmatics and poor mind-reading. Psychol. Med..

[18] Addington J, Penn D, Woods SW, Addington D, Perkins DO (2008). Facial affect recognition in individuals at clinical high risk for psychosis. Br. J. Psychiatry.

[19] Chung YS, Kang DH, Shin NY, Yoo SY, Kwon JS (2008). Deficit of theory of mind in individuals at ultra-high-risk for schizophrenia. Schizophr. Res..

[20] Guastella AJ (2013). Social cognitive performance as a marker of positive psychotic symptoms in young people seeking help for mental health problems. Schizophr. Res..

[21] Brune M, Abdel-Hamid M, Lehmkamper C, Sonntag C (2007). Mental state attribution, neurocognitive functioning, and psychopathology: what predicts poor social competence in schizophrenia best?. Schizophr. Res..

[22] Nuske HJ, Vivanti G, Dissanayake C (2013). Are emotion impairments unique to, universal, or specific in autism spectrum disorder? A comprehensive review. Cogn. Emot..

[23] Losh M (2009). Neuropsychological profile of autism and the broad autism phenotype. Arch. Gen. Psychiatry.

[24] Constantino JN (2017). Infant viewing of social scenes is under genetic control and is atypical in autism. Nature.

[25] Cotter J (2018). Social cognitive dysfunction as a clinical marker: a systematic review of meta-analyses across 30 clinical conditions. Neurosci. Biobehav. Rev..

[26] Rutherford MD, Baron-Cohen S, Wheelwright S (2002). Reading the mind in the voice: a study with normal adults and adults with Asperger syndrome and high functioning autism. J. Autism Dev. Disord..

[27] Morrison KE (2017). Distinct profiles of social skill in adults with autism spectrum disorder and schizophrenia. Autism Res..

[28] Lee RSC (2017). A transdiagnostic study of education, employment, and training outcomes in young people with mental illness. Psychol. Med..

[29] Craig JS, Hatton C, Craig FB, Bentall RP (2004). Persecutory beliefs, attributions and theory of mind: comparison of patients with paranoid delusions, Asperger’s syndrome and healthy controls. Schizophr. Res..

[30] Couture SM (2010). Comparison of social cognitive functioning in schizophrenia and high functioning autism: more convergence than divergence. Psychol. Med..

[31] Bolte S, Poustka F (2003). The recognition of facial affect in autistic and schizophrenic subjects and their first-degree relatives. Psychol. Med..

[32] Martinez G (2017). Phenotypic continuum between autism and schizophrenia: Evidence from the Movie for the Assessment of Social Cognition (MASC). Schizophr. Res..

[33] Couture SM, Penn DL, Roberts DL (2006). The functional significance of social cognition in schizophrenia: a review. Schizophr. Bull..

[34] Plana I, Lavoie MA, Battaglia M, Achim AM (2014). A meta-analysis and scoping review of social cognition performance in social phobia, posttraumatic stress disorder and other anxiety disorders. J. Anxiety Disord..

[35] Lord C (2012). Autism Diagnostic Observation Schedule (ADOS-2).

[36] Brown TA, Barlow DH (2014). Anxiety and Related Disorders Interview Schedule for DSM-5 (ADIS-5)..

[37] Brown TA, DiNardo PA, Barlow DH (1994). Anxiety Disorders Interview Schedule for DSM-IV.

[38] First MB, Spitzer RL, Gibbon M, Williams JBW (1995). Structured Clinical Interview for DSM-IV Axis I Disorders — Patient Edition (SCID-I/P, Version 2.0), Biometrics Research.

[39] Wechsler D (1999). Wechsler Abbreviated Scale of Intelligence (WASI) Manual.

[40] Wechsler D (2001). Wechsler Test of Adult Reading: WTAR.

[41] Lovibond PF, Lovibond SH (1995). The structure of negative emotional states: comparison of the Depression Anxiety Stress Scales (DASS) with the Beck Depression and Anxiety Inventories. Behav. Res. Ther..

[42] Heimberg RG, Mueller GP, Holt CS, Hope DA, Liebowitz MR (1992). Assessment of anxiety in social interaction and being observed by others: the social interaction anxiety scale and the Social Phobia Scale. Behav. Ther..

[43] Allison C, Auyeung B, Baron-Cohen S (2012). Toward brief “Red Flags” for autism screening: the short autism spectrum quotient and the short quantitative checklist for autism in toddlers in 1,000 cases and 3,000 controls [corrected]. J. Am. Acad. Child Adolesc. Psychiatry.

[44] Baron-Cohen S, Wheelwright S, Hill J, Raste Y, Plumb I (2001). The “Reading the Mind in the Eyes” Test revised version: a study with normal adults, and adults with Asperger syndrome or high-functioning autism. J. Child Psychol. Psychiatry.

[45] Young A, Perrett D, Calder A, Sprengelmeyer R, Ekman P (2002). Facial Expressions of Emotion: Stimuli and Tests (FEEST).

[46] Adolphs R, Tranel D (2003). Amygdala damage impairs emotion recognition from scenes only when they contain facial expressions. Neuropsychologia.

[47] Stone VE, Baron-Cohen S, Knight RT (1998). Frontal lobe contributions to theory of mind. J. Cogn. Neurosci..

[48] Langdon R (1997). Defective self and/or other mentalising in Schizophrenia: a cognitive neuropsychological approach. Cogn. Neuropsychiatry.

[49] Baron-Cohen S, Wheelwright S (2004). The empathy quotient: an investigation of adults with Asperger syndrome or high functioning autism, and normal sex differences. J. Autism Dev. Disord..

[50] Muncer SJ, Ling J (2006). Psychometric analysis of the empathy quotient (EQ) scale. Pers. Individ. Dif..

[51] Ustun TB (2010). Developing the World Health Organization Disability Assessment Schedule 2.0. Bull. World Health Organ..

[52] Croca M (2018). Theory of mind and schizophrenia in young and middle-aged patients: Influence of executive functions. Psychiatry Res..

[53] Rojas DC (2006). Regional gray matter volumetric changes in autism associated with social and repetitive behavior symptoms. BMC Psychiatry.

[54] Hahamy A, Behrmann M, Malach R (2015). The idiosyncratic brain: distortion of spontaneous connectivity patterns in autism spectrum disorder. Nat. Neurosci..

[55] Buchy L (2017). Mapping structural covariance networks of facial emotion recognition in early psychosis: a pilot study. Schizophr. Res..

[56] Goldin PR (2013). Impact of cognitive behavioral therapy for social anxiety disorder on the neural dynamics of cognitive re-appraisal of negative self-beliefs. JAMA Psychiatry.

[57] Clarke PJ, Hickie IB, Scott E, Guastella AJ (2012). Clinical staging model applied to young people presenting with social anxiety. Early Interv. Psychiatry.

