# Supplementary material for: Autism, early psychosis, and social anxiety disorder: understanding the role of social cognition and its relationship to disability in young adults with disorders characterized by social impairments
Source: Transl Psychiatry. 2018 Oct 26;8:233. doi: 10.1038/s41398-018-0282-8 (PMC6203776; doi:10.1038/s41398-018-0282-8)
Supplement: Supplementary file 1 — Supplementary Information [file 41398_2018_282_MOESM1_ESM.docx]

**Supplementary Table 1.** Summary of measures used.

| **Measure** | **Domain** | **Description** | **Outcome measures** |
| --- | --- | --- | --- |
| **Autism Diagnostic Observation Schedule – 2nd edition**  **(ADOS-2** | Diagnostic  (Autism Spectrum Disorder) | Standardised assessment for suspected autism spectrum disorder, including social interaction and communication deficits and restricted and repetitive behaviours. | A high score indicates a diagnosis of autism spectrum disorder. |
| **Anxiety Diagnostic Interview Schedule IV/5**  **(ADIS-IV/5)** | Diagnostic (SocialAnxiety Disorder) | Structured assessment interview for suspected anxiety disorders, based on DSM-IV or DSM-5 criteria. | Meeting the DSM criteria in the Social Anxiety Disorder section of this assessment indicates a diagnosis of social anxiety disorder. |
| **Structured Clinical Interview for DSM-IV Axis I Disorders**  **(SCID-I)** | Diagnostic (First Episode Psychosis) | Structured assessment interview for DSM-IV Axis I diagnosis. | Meeting the DSM criteria for a diagnosis of a psychotic disorder. |
| **Wechsler Test of Adult Reading**  **(WTAR)** | Intelligence (IQ) | Assessment of IQ, in which the participant reads aloud a list of 50 words. Correct readings are scored, and an estimate of full-scale IQ is derived from the total score. | A higher score indicates higher intelligence. |
| **Wechsler Abbreviated Scale of Intelligence (WASI)** | Intelligence (IQ) | Assessment of IQ, in which the participant completes the vocabulary and matrix reasoning subscales. . An estimate of full-scale IQ is derived from the total scores of these subscales. | A higher score indicates higher intelligence. |
| **Reading the Mind in the Eyes Test (RMET)** | Emotion Recognition | For this test, participants were presented with photographs of human eyes and were then required to nominate which emotion was being expressed in the photograph from a choice of four emotions. A total score was calculated based on the accuracy of their responses. Higher scores reflect higher levels of social cognition. 36 images displaying the eye region of human faces and depict various emotional expressions. The participant is asked to pick which of four words best describes what the person in the photo is thinking or feeling. RMET yields a total score of correct answers. | A total score was calculated based on the accuracy of their responses. Higher scores reflect higher levels of social cognition. |
| **Facial Expressions of Emotions: Stimuli and Tests (FEEST)** | Emotion Recognition | A set of tests of facial affect identification. We will use the Ekman 60 Faces Test, in which a total of 60 photos of faces are presented in a random order for five seconds each, with 10 photos for each of the six basic emotions (happiness, surprise, fear, sadness, disgust, anger). | A total score was calculated based on the accuracy of their responses. Higher scores reflect higher levels of social cognition. |
| **Movie Stills task** | Emotion Recognition | This task consists of 16 photographs of complex scenes from movies with clear emotional content. Participants are first shown the movie stills with the faces blocked out, and then are shown the 16 photographs with the faces present. Comparison of stimuli with and without faces assesses emotion recognition from purely contextual cues versus using both facial expressions and contextual cues to determine emotion. Participants choose one of seven emotion words (happy, sad, afraid, surprised, angry, disgusted, or neutral) that best describe what the actors in the movie still are feeling. Performance is converted to accuracy scores. | Total scores for the “no face” and “face” subtests were calculated based on the accuracy of their responses. Higher scores reflect higher levels of social cognition. |
| **False-Belief Picture Sequencing Task (FBPST)** | Theory of Mind | Participants are shown four-picture cards that must be sequenced correctly to tell a story. There are four types of stories. “False Belief” stories require an understanding that a story character has acted on a false belief, a classic index of Theory of Mind ability. “Social Script” require an understanding of appropriate conventional social behaviours. “Capture” stories require the ability to focus attention on only relevant factors in the story. “Mechanical” stories require an understanding of physical cause and effect. The first two sets of stories measure social cognition, while the letter two sets of stories serve as controls for general picture-sequencing ability. 16 stories are completed all together. | Total scores for the “false belief”, “social script”, “capture” and “mechanical” subtests were calculated based on the accuracy of their responses. Higher scores reflect higher levels of the relevant type of cognition. |
| **Faux Pas Recognition Task** | Theory of mind | This task involves the researcher reading 10 brief stories to the research participant and then to asking the participant whether someone in the story said something that they should not have said. 5 of the stories involve socially awkward situations (“faux pas”), and the other 5 do not (“non faux pas). The participant is also asked additional questions so that the experimenter can confirm that the research subject understands why the faux pas comment should not have been made and why the person who made the faux pas comment might have said it. | For this project, the proportion of “Faux Pas” stories correctly identified as such was calculated to produce the Faux Pas Hit Rate score. The proportion of “Non Faux Pas” incorrectly identified as having socially awkward content was calculated to produce the Faux Pas False Alarm Rate score. A high Hit Rate and low False Alarm rate reflects a higher level of social cognition. |
| **Cambridge Behaviour Scale Abbreviated Empathy Quotient (EQ) Scale** | Empathy (self-report) | Self-report rating of whether a participant engages in either affective empathy (e.g., “Seeing other people cry does not really upset me”) or cognitive empathy (e.g., “I can easily work out what another person might want to talk about”) in social situations or relationships. This is an abbreviated version of the standard EQ scale with only 15 items. | A high total EQ score reflects a higher level of social cognition. |
| **Autism Quotient – short form**  **(AQ-10)** | Autism (self-report) | Self-report rating of whether a participant has autistic traits and behaviours. This is an abbreviated version of the standard AQ scale with only 10 items. | A high total AQ score reflects a higher level of autistic traits and behaviours. |
| **Social Interaction Anxiety Scale (SIAS)** | Social Anxiety  (self-report) | A questionnaire in which participants rate how much anxiety they typically experience in a range of social interactions. | A higher score reflects higher levels of social anxiety. |
| **Depression Anxiety Stress Scale (DASS-21)** | Depression, anxiety & stress (self-report) | A questionnaire in which participants rate how much a series of statements that reflect depression, anxiety and stress apply to themselves. | Separate total scores for depression, anxiety and stress items are calculated, but only the Depression scores were used in this study. High scores indicate high levels of depression. |
| **World Health Organisation Disability Assessment Schedule 2 (WHODAS-2.0),** | Disability (self-report) | A questionnaire that asks participants to rate how much difficulty they experience in a variety of everyday circumstances grouped into six domains, including understanding and communicating, getting around, self care, getting along with people, life activities (home/school/work), and participation in society. | Total disability scores were calculated for each of these domains. Higher scores indicate higher levels of disability. The 32-item Total Disability score and the “Getting Along With People” domain score were used in this study. |

**Supplementary Table 2.** Summary of missing data for groups. Figures represent number of participants who are missing each measure in each of the diagnostic groups.

| **Measure** | **Autism Spectrum Disorder**  **(N = 53)** | **Early Psychosis**  **(N = 51)** | **Social Anxiety Disorder**  **(N = 64)** | **Neurotypical Controls**  **(N = 31)** |
| --- | --- | --- | --- | --- |
| Reading the Mind in the Eyes Test (RMET) | 0 | 0 | 1 | 0 |
| Facial Expressions of Emotions: Stimuli and Tests (FEEST) | 3 | 1 | 0 | 1 |
| Movie Stills task | 2 | 0 | 2 | 0 |
| False-Belief Picture Sequencing Task (FBPST) | 0 | 0 | 0 | 0 |
| Faux Pas Recognition Task | 1 | 0 | 4 | 0 |
| Cambridge Behaviour Scale Abbreviated Empathy Quotient (EQ) Scale | 5 | 0 | 6 | 6 |
| Social Interaction Anxiety Scale (SIAS) | 0 | 0 | 0 | 0 |
| Depression Anxiety Stress Scale (DASS-21) | 0 | 0 | 0 | 0 |
| World Health Organisation Disability Assessment Schedule 2 (WHODAS-2.0), | 0 | 14 | 1 | 0 |

**Supplementary Table 3.**  Summary of psychotropic medication use for diagnostic groups. Figures represent number of participants taking each type of medication in each of the diagnostic groups.

| **Medication Type** | **Autism Spectrum Disorder**  **(N = 53)** | **Early Psychosis**  **(N = 51)** | **Social Anxiety Disorder**  **(N = 64)** |
| --- | --- | --- | --- |
| Antidepressants | 21 (40%) | 14 (28%) | 24 (38%) |
| Sedatives/Hypnotics | 1 (2%) | 0 (0%) | 1 (2%) |
| Mood Stabilisers | 4 (8%) | 9 (18%) | 3 (5%) |
| Antipsychotics | 12 (23%) | 43 (84%) | 4 (6%) |
| Benzodiazepines | 1 (2%) | 2 (4%) | 4 (6%) |
| Stimulants | 10 (19%) | 0 (0%) | 2 (3%) |
| Anticonvulsants | 0 (0%) | 0 (0%) | 1 (2%) |

**Supplementary Table 4.**  Summary of alcohol, tobacco and recreational drug use within the last 3 months for diagnostic groups. Figures represent the number (and percentage) of participants using each type of substance in each of the diagnostic groups.

| **Drug Type** | **Autism Spectrum Disorder**  **(N = 53)** | **Early Psychosis**  **(N = 51)** | **Social Anxiety Disorder**  **(N = 64)** | **Neurotypical Control**  **(N = 31)** |
| --- | --- | --- | --- | --- |
| Tobacco | 6 (11%) | 18 (35%) | 11 (17%) | 5 (16%) |
| Alcohol | 26 (49%) | 26 (51%) | 32 (50%) | 21 (68%) |
| Cannabis | 4 (8%) | 10 (20%) | 10 (16%) | 2 (6%) |
| Cocaine | 1 (2%) | 3 (6%) | 1 (2%) | 0 (0%) |
| Inhalants | 1 (2%) | 1 (2%) | 3 (5%) | 0 (0%) |
| Sedatives | 4 (8%) | 6 (12%) | 9 (14%) | 0 (0%) |
| Hallucinogens | 1 (2%) | 1 (2%) | 1 (2%) | 1 (3%) |
| Opioids | 2 (4%) | 1 (2%) | 2 (3%) | 0 (0%) |
| Other | 0 (0%) | 1 (2%) | 2 (3%) | 0 (0%) |
